# Reliability and validity of the practice environment scale of the nursing work index for Japanese hospital nurses

**DOI:** 10.1002/nop2.148

**Published:** 2018-05-18

**Authors:** Yasuko Ogata, Miki Sasaki, Yoshie Yumoto, Yuki Yonekura, Midori Nagano, Katsuya Kanda

**Affiliations:** ^1^ Department of Gerontological Nursing and Care System Development Graduate School of Health Care Sciences Tokyo Medical and Dental University (TMDU) Bunkyo‐ku Tokyo Japan; ^2^ Graduate School of Nursing Science St. Luke's International University Chuo‐ku Tokyo Japan; ^3^ Department of Adult Nursing School of Nursing The Jikei University Chofu‐shi Japan; ^4^ Faculty of Health Science Aino University Ibaraki‐shi Osaka Japan

**Keywords:** Japan, magnet hospital, nursing, PES‐NWI, reliability, validity

## Abstract

**Aims:**

The aim of this study was to examine the reliability and validity of the Practice Environment Scale of the Nursing Work Index (PES‐NWI) for hospital nurses in Japan.

**Design:**

A cross‐sectional mail survey.

**Methods:**

Participants in this study were 1,219 full‐time ward nurses from 27 hospitals in Japan, using 31 items of the Japanese version of the PES‐NWI questionnaire, from December 2008‐March 2009. Construct validity, criterion‐related validity and internal consistency of the PES‐NWI were tested.

**Results:**

The PES‐NWI showed reliable internal consistency. The five‐factor structure was supported by confirmatory factor analysis. The PES‐NWI correlated significantly with job satisfaction, burnout and the nurses’ intention to stay on the job, supporting criterion‐related validity.

## 1 INTRODUCTION

Like other countries, Japan is experiencing a nurse shortage at the same time its rapidly ageing population demands increasing nursing services for the older people. According to the International Council of Nursing, the combination of “recruitment and retention” and “workforce planning” is known to be an effective policy‐based intervention framework (Buchan & Calman, 2005). To attract nurses to the workplace, the features of the practice environment are important. To quantify those features, useful instruments have been developed in the United States, such as the Nursing Work Index (NWI), the Revised Nursing Work Index (NWI‐R) and the Practice Environment Scale of the Nursing Work Index (PES‐NWI). These are based on the features of so‐called “magnet hospitals,” which, during a nursing shortage in the early 1980, were, like magnets, able to attract and retain well‐qualified nurses, while consistently providing high‐quality care.

The translated instruments should be useful in attracting nurses to high‐quality hospitals by comparing the features of the practice environment among hospitals, both domestically and internationally. This paper presents the results of a national study in Japan that corroborated the reliability and validity of the PES‐NWI in Japanese.

## 2 BACKGROUND

The PES‐NWI, developed by Lake (2002) as one of the instruments to examine the practice environment for nurses, was based on the NWI. In the PES‐NWI, “nursing practice environment” was defined as “the organizational characteristics of a work setting that facilitate or constrain professional nursing practice” (Lake, 2002, p. 178). It has been endorsed as a gauge of the quality of the nursing practice environment by several organizations in the USA, such as the National Quality Forum (NQF) (Warshawsky & Havens, 2011, p. 18). The PES‐NWI consists of five subscales, which are “Nurse Participation in Hospital Affairs,” “Nursing Foundations for Quality of Care,” “Nurse Manager Ability, Leadership and Support of Nurses,” “Staffing and Resource Adequacy,” and “Collegial Nurse‐Physician Relations” (Lake, 2002, p. 181). The five subscales have been shown to have acceptable internal consistency and reliability.

The PES‐NWI has been translated into many languages, such as French, Chinese and Icelandic (Warshawsky & Havens, 2011). The PES‐NWI has been, in many countries, examined for its relationship with other variables that reflect “nurse outcomes,” such as job satisfaction, intent to leave job, empowerment and burnout level; “patient outcomes,” such as mortality, nurse‐rated quality of care and hospitalization; and “organizational variables,” such as turnover rate (Warshawsky & Havens, 2011). Because items of the PES‐NWI reflect the features of “magnet hospitals,” it was expected that higher scores of the PES‐NWI would lead to higher staff‐nurse job satisfaction and retention of staff nurses and lower burnout level. Manojlovich (2005) identified, using the answers of 332 respondents from a random sample of 500 hospital nurses in Michigan, factors in the practice environment that contribute to nurses’ job satisfaction. In Canada, “practice environment” had a positive correlation with personal accomplishment and a negative one with emotional exhaustion (Leiter & Laschinger, 2006). A study of Registered Nurses (RNs) in US‐based Army Medical Department hospitals showed that an unfavourable nursing practice environment was significantly correlated with job dissatisfaction, emotional exhaustion, intent to leave and poor quality of care (Patrician, Shang, & Lake, 2010).

The PES‐NWI was translated into Japanese with both a forward translation and back‐translation process, which included a method for confirming the equivalence between the original items of the PES‐NWI and the items of the backward‐translated version (Ogata, Nagano, & Akanuma, 2008). Since the PES‐NWI translations have been available, it has continued to be used by researchers (Anzai, Douglas, & Bonner, 2014; Narita & Ishii, 2015; Ogata, Nagano, Fukuda, & Hashimoto, 2011; Ogata et al., 2010; Tei‐Tominaga, Tsuchiya, & Sato, 2012), as well as by nurse administrators. This phenomenon indicates that an instrument that measures the nursing practice environment is also needed in Japan. In a preliminary study for reliability and validity testing that used data from 282 RNs in six Japanese hospitals, test‐retest reliability and internal consistency were found to be acceptable (Ogata et al., 2010). However, proving construct validity requires a study of the PES‐NWI for a larger sample (Ogata et al., 2010). For international comparisons, confirmation of the reliability and validity of the original items, without modification, is needed. The aim of this study was to confirm the reliability and validity of the Japanese version of the PES‐NWI with a larger sample than was used in the preliminary study.

## 3 THE STUDY

### 3.1 Method

This study is cross‐sectional and consists of reliability and validity testing with data obtained from a mail survey.

### 3.2 Participants

Invitations to nurse participants occurred in two steps. As the first step, 27 chief nursing officers out of the 696 candidate hospitals whose number of beds exceeded 50 notified us that their hospital would participate in the mail survey. The 696 hospitals were located in Japanese cities whose population is more than 500,000, such as Tokyo, Osaka and Kyoto. University hospitals were excluded, because their functions, which include education and research in addition to medical care, were different in kind. As the second step, 4,688 ward nurses from the 27 responding hospitals were delivered anonymous self‐reported questionnaires from December 2008‐March 2009. In this study, we considered only the questionnaires returned by full‐time nurses.

### 3.3 Measures

The self‐reported questionnaire to nurses included the following: (1) demographic and professional experience; (2) the PES‐NWI (Lake, 2002) translated into Japanese (PES‐NWI‐J); (3) the Work Satisfaction Instrument in Japanese (WSI‐J) (Ozaki & Tadamasa, 1988); (4) the Japanese Burnout Scale (JBS) (Kubo & Tao, 1994; Tao & Kubo, 1996) and (5) an original item, “Intention to remain in or leave the workplace next year.”

The demographic and professional experience of participants included gender, age, educational background, years of nursing practice experience and marital status. The PES‐NWI items included the following instruction (Lake, 2002): “For each item, please indicate the extent to which you agree that the item is PRESENT IN YOUR CURRENT JOB. Indicate your degree of agreement by circling the appropriate number.” The four response categories (Lake, 2002) were from “strongly agree (=1)”—“strongly disagree (=4).” The subscales were calculated as mean scores after the reply values were reversed; for example, “strongly agree” became 4 and “strongly disagree became 1. The composite was calculated as the mean of the five subscale scores.

To confirm the criterion‐related validity of the PES‐NWI‐J, three instruments were used. First, the Work Satisfaction Instrument (Stamps, Piedmont, Slavitt, & Haase, 1978) translated into Japanese, namely WSI‐J, was applied, because the organizational characteristics of “magnet hospitals” were originally related to job satisfaction for nurses (McClure, Poulin, Sovie, & Wandelt, 1983). In previous studies, the PES‐NWI scores also had a positive relationship with job satisfaction (Gabriel, Erickson, Moran, Diefendorff, & Bromley, 2013; Manojlovich, 2005; Manojlovich & Laschinger, 2007) or a negative relationship with job dissatisfaction (Liu et al., 2012). As the WSI‐J has been used in many previous studies in Japanese nursing settings (Kubo et al., 2007; Nakagaki, 2011; Ueno, Syoji, Setsuko, Takashima, & Takama, 1999) and has been confirmed for its reliability and validity (Ozaki & Tadamasa, 1988), WSI‐J was applied in this study. The Cronbach's alpha coefficient was 0.89 for the WSI‐J in this study.

Second, the JBS was used for criterion‐related validity testing, because the original PES‐NWI scores were reported to have negative associations with nurse burnout (Gabriel et al., 2013; Hanrahan, Aiken, McClaine, & Hanlon, 2010; Laschinger & Leiter, 2006; Leiter & Laschinger, 2006). The JBS was developed based on the setting of a Japanese human‐services organization, including nursing units (Kubo & Tao, 1994). It consists of 17 items with three sub‐dimensions that are the same as the Maslach Burnout Inventory (MBI), which was developed to assess three aspects of the burnout syndrome (Maslach & Jackson, 1981), namely, emotional exhaustion (EE), depersonalization (DP) and personal accomplishment (PA) (Tao & Kubo, 1996). The minimum and maximum values for the subscale of the JBS are 5 and 25 for EE, 6 and 30 for DP and 6 and 30 for PA. Higher EE scores and DP scores and lower PA scores, indicate a more serious burnout situation. The Cronbach's alpha coefficients measured by this study were 0.83 for EE, 0.84 for DP and 0.78 for PA.

Finally, an original item, “Intention to remain in or leave the hospital next year,” with four answer options from “remain” to “resign,” was used. In this study, “remain” and “probably remain” were interpreted as “intention to remain at the hospital in the next year,” while “probably resign” and “resign” were interpreted as “intention to resign from the hospital within the next year.”

### 3.4 Analysis

Descriptive statistics were calculated regarding 27 hospital characteristics, nurses’ characteristics, the PES‐NWI‐J scores and the other variables. To assess reliability of the PES‐NWI‐J, internal consistency, namely, Cronbach's alpha coefficients of the five subscales and the composite, were calculated. To assess construct validity, confirmatory factor analysis was conducted to determine whether the data of the participants would show the same structure as the 5‐factor model theorized in the original PES‐NWI. Model fitness was assessed with goodness‐of‐fit index (GFI), normed fit index (NFI), comparative fit index (CFI) and root‐mean‐square error of approximation (RMSEA). The model was built using the five subscales of the original PES‐NWI.

To explore the criterion‐related validity, we examined the relationships between the scores of the PES‐NWI‐J and WSI‐J or JBS scores, using nurses’ age as a control variable because satisfaction (Kubo et al., 2007) and burnout (Motomura & Yatsushiro, 2010) were related to respondents’ age. The relationships between the PES‐NWI‐J scores and “Intention to remain in or leave the hospital next year” were also tested by logistic regression analysis, with the latter as the dependent variable and the composite and subscale scores of the PES‐NWI‐J and “nurse's age” as the independent variables. Nurse's age was used as a control variable in logistic regression models. All statistical analyses were done using SPSS Statistics version 23.0 and AMOS version 23.0. A *p*‐value of .05 was used as the cut‐off for significance.

### 3.5 Ethics

Study protocol and the process were reviewed and approved by the Ethics Committee of the Graduate School of Nursing, Chiba University, Japan. The return of the anonymous self‐reported questionnaire from ward nurses in the mail survey was taken as the consent of participation by the nurses.

## 4 RESULTS

### 4.1 Characteristics of hospitals and participants

Regarding the 27 hospitals’ characteristics, the mean for number of beds was 311.7 beds (*SD* 159.3) and average length of stay was 16.9 days (*SD* 4.9). As for the fee category, which in Japan is based on the ratio of patients to nurses, the highest category, “seven‐to‐one,” comprised 15 hospitals (55.6%) and the second highest, “ten‐to‐one,” comprised nine hospitals (33.3%).

Among 1,847 survey respondents, the 1,219 participants’ answers that had no missing values for the variables used in the analysis to confirm the reliability and validity were considered. Their average age was 31.8 years old (*SD* 7.8) and the average amount of nursing experience was 9.3 years (*SD* 7.2). The average of their work experience at the current hospital was 6.3 years (*SD* 6.2). All of them are full‐time nurses, not part‐time. Of the 1,219 participants, 97.4% were female (*N* = 1,187) and 30.4% were married (*N* = 371). As for educational background, 80.5% (*N* = 981) finished nursing school only and 6.1% (*N* = 75) had achieved a BA or higher degree.

### 4.2 Descriptive results

The average scores of the PES‐NWI were 2.47 (*SD* 0.37) for Composite, 2.50 (*SD* 0.41) for “Nurse Participation in Hospital Affairs,” 2.55 (*SD* 0.39) for “Nursing Foundations for Quality of Care,” 2.69 (*SD* 0.59) for “Nurse Manager Ability, Leadership and Support of Nurses,” 2.13 (*SD* 0.54) for “Staffing and Resource Adequacy,” and 2.50 (*SD* 0.56) for “Collegial Nurse‐Physician Relations.” Mean of nurses’ satisfaction, namely, WSI‐J, was 138.3 (*SD* 26.5). Burnout scores were 16.90 (*SD* 4.42) for EE, 13.02 (*SD* 4.71) for DP and 14.10 (*SD* 4.15) for PA. As for intention to remain or leave, 22.5% (*N* = 274) of the nurses intended to resign from their hospital in the next year.

### 4.3 Reliability and validity testing

The Cronbach's alpha coefficients were 0.78 for “Nurse Participation in Hospital Affairs,” “Nursing Foundations for Quality of Care,” and “Staffing and Resource Adequacy,” 0.86 for “Nurse Manager Ability, Leadership and Support of Nurses,” 0.81 for “Collegial Nurse‐Physician Relations,” and 0.79 for the composite.

**Figure 1:**
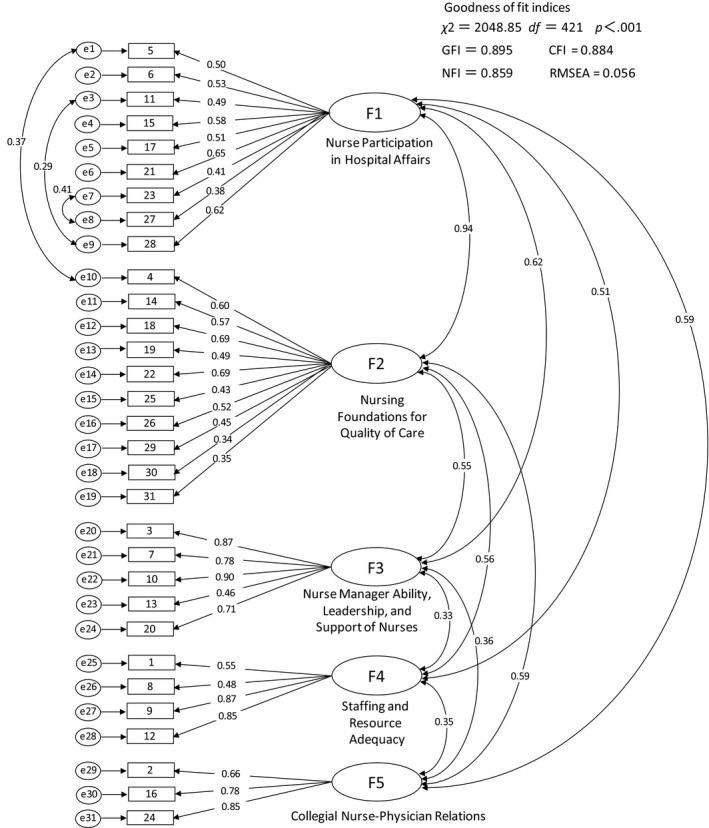
Five‐factor latent measurement model for the PES‐NWI in Japanese, with standardized regression weights and correlations

The model of the confirmatory factor analysis was built using the five subscales of the original PES‐NWI (Figure 1). All path coefficients were significant. The chi‐square value was also significant (χ^2 ^= 2048.85, *df* = 421, *p* < .001). The goodness‐of‐fit indices of GFI, CFI, NFI and RMSEA were 0.895, 0.884, 0.859 and 0.056, respectively. Regarding the relationships between the structure's latent variables, the strongest occurred between factors 1 (Nurse Participation in Hospital Affairs) and 2 (Nursing Foundations for Quality of Care), which yielded a value of 0.94. Before the consideration of error coefficients for three pairs of items shown in Figure 1, the goodness‐of‐fit indices of the model were 0.868, 0.850, 0.825 and 0.064 for of GFI, CFI, NFI and RMSEA respectively.

Criterion‐related validity was tested as the relationships between the PES‐NWI‐J and the WSI‐J, the JBS and “Intention to remain in or leave the workplace.” The composite of the PES‐NWI‐J indicated statistically significant positive correlation with the WSI‐J (*r* = .75, *p* < .001) (Table 1). The composite also showed statistically significant, negative correlation with EE (*r* = −.38, *p* < .001) and DP (*r* = −.40, *p* < .001) of the JBS and positive correlation with PA of the JBS (*r* = .28, *p* < .001) (Table 1).

**Table 1:** Partial correlation coefficients between composite of the PES‐NWI in Japanese and WSI‐J or JBS

		PES‐NWI (Composite)
WSI‐J		0.75*
JBS	EE	−0.38*
	DP	−0.40*
	PA	0.28*

WSI‐J, Work Satisfaction Instrument in Japanese; JBS, Japanese Burnout Scale; EE, emotional exhaustion; DP, depersonalization; PA, personal accomplishment.

*p <* *.001.

Participants’ intention to remain in (=1) or leave (=0) the hospitals related significantly to nurses’ age: younger groups (less than 25 years old, 26–29 years old, 30–34 years old and 35–39 years old) tended to leave more readily than the elder group (40 years old and over) (*p* < .001). In the logistic regression analysis (Table 2), the composite score of the PES‐NWI‐J (Odds Ratio: OR = 4.99, 95% Confidence Interval: CI = 3.36–7.40; *p* < .001) related significantly to the nurses’ intention to remain. All of the five subscale scores also related significantly to the nurses’ intention to remain: (OR = 3.59, 95% CI = 2.55–5.05; *p* < .001) for “Nurse Participation in Hospital Affairs,” (OR = 3.78, 95% CI = 2.63–5.43; *p* < .001) for “Nursing Foundations for Quality of Care,” (OR = 2.06, 95% CI = 1.63–2.59; *p* < .001) for “Nurse Manager Ability, Leadership and Support of Nurses,” (OR = 2.55, 95% CI = 1.94–3.35; *p* < .001) for “Staffing and Resource Adequacy,” and (OR = 1.58, 95% CI = 1.24–2.02; *p* < .001) for “Collegial Nurse‐Physician Relations.” Higher scores of the PES‐NWI related to job retention of staff nurses.

**Table 2:** Relationship between composite of the PES‐NWI in Japanese and intention to remain for nurse participants

Explanatory variables	Odds Ratio (95% Confidence Interval)
PES‐NWI	
Composite	4.99 (3.36–7.40)*
Subsacles:	
Nurse Participation in Hospital Affairs	3.59 (2.55–5.05)*
Nursing Foundations for Quality of Care	3.78 (2.63–5.43)*
Nurse Manager Ability, Leadership, and Support of Nurses	2.06 (1.63–2.59)*
Staffing and Resource Adequacy	2.55 (1.94–3.35)*
Collegial Nurse‐Physician Relations	1.58 (1.24–2.02)*

Adjusted for age groups (less than 25 years old, 26–29 years old, 30–34 years old, 35–39 years old, and 40 years old and over).

*p <* *.001.

## 5 DISCUSSION

The PES‐NWI in Japanese (PES‐NWI‐J) had already been confirmed for its equivalence with the original items of the PES‐NWI (Ogata et al., 2008). This study confirmed that it was valid and reliable in a Japanese context with a large sample. As a result, the PES‐NWI‐J may be used in future studies to ascertain the nursing practice environment in Japan.

### 5.1 Reliability

Internal consistency of the PES‐NWI‐J was confirmed, because Cronbach's alphas of the PES‐NWI‐J in this study, from 0.78 to 0.86, were acceptable for both the five subscales and the composite. Compared with the Cronbach's alphas found in previous studies that used the PES‐NWI translated into other languages, they demonstrate satisfactory reliability of the PES‐NWI‐J; Cronbach's alpha were 0.71–0.84 in the original PES‐NWI (Lake, 2002), 0.75–0.92 (Anzai et al., 2014; Ogata et al., 2011) in Japanese, 0.65–0.90 in Chinese (Chiang & Lin, 2009) and 0.73–0.90 in Spanish (Fuentelsaz‐Gallego, Moreno‐Casbas, & Gonzalez‐Maria, 2013).

### 5.2 Validity

In this study, validity was confirmed as “construct validity” founded on confirmatory factor analysis and as “criterion‐related validity” based on the relations between the PES‐NWI and the other variables.

#### 5.2.1 Construct validity

Because the chi‐square test is affected by sample size, the model is easily rejected when the sample size is large (Hooper, Coughlan, & Mullen, 2008; Toyoda, 2011), as in this study. Therefore, goodness‐of‐fit in the confirmatory factor analysis has been comprehensively assessed by multiple indices. GFI (Toyoda, 2011) and RMSEA (Steiger, 2007) supported the model fit, although chi‐square, CFI and NFI were marginal.

Goodness‐of‐fit indices of the model were improved by considering correlated errors for three pairs of items in Figure 1. The phenomenon was potentially caused by the correlation between factors 1 and 2 in Figure 1, namely, the subscales 1 and 2 of the PES‐NWI, respectively. The correlation coefficients between those two factors were high compared with the relationships among the other factors in previous studies, such as 0.65 (Lake, 2002) and 0.74 (Hanrahan et al., 2010). The relationships among the structure's latent variables in the two factors were largest in both models used in this study, with trends the same as with the PES‐NWI in Spanish (Pedro‐Gómez et al., 2012). Therefore, the relationship between the two factors might be relatively greater in this study. In the context of this relationship, it is possible that the meanings of the items considered “correlated errors” are also similar. Although the two items that represent one of the three pairs considered correlated errors belonged to two different factors, their meanings were similar. Specifically, “item 5: Career development/clinical ladder opportunity” in factor 1 and “item 4: Active staff development or continuing education programs for nurses” in factor 2, both reflect staff development in the organization.

The goodness‐of‐fit of the confirmatory factor analysis can be improved by participant and organizational characteristics, too. For instance, although Pedro‐Gómez et al. (2012) showed a better CFI (0.964) in the Spanish version than the scores in this study (0.884) and in a Korean study (0.85 & 0.86) (Cho, Choi, Kim, Yoo, & Lee, 2011), a big difference among these was participants’ age: 44.5 years old in the Spanish study, but 31.8 in this study and 28.4 in the Korean study. In general, as nurses gain experience, managerial work is added to nursing practice. There is a possibility that the evaluation of practice environment differs between more experienced and less experienced, younger nurses. Concerning organizational characteristics, the composite scores differed by type of organization: 2.67 for Public sector, 2.70 for Private sector and 2.51 for Aged‐care sector (Parker, Tuckett, Eley, & Hegney, 2010). Thus, future research might consider participants’ “age mix” or “nurse‐experience mix” and the different characteristics of organizational types.

#### 5.2.2 Criterion‐related validity

Criterion‐related validity testing showed that the PES‐NWI composite score had relatively high correlations with the WSI‐J, medium negative correlations with EE and DP and positive correlations with PA. The better that the PES‐NWI composite score was, the more that nurses were satisfied, as in a previous study (Manojlovich, 2005). Better PES‐NWI composite scores also correlated with lower burnout scores, as in previous studies (Aiken, Clarke, Sloane, Lake, & Cheney, 2008; Liu et al., 2012).

PES‐NWI scores related significantly to the nurses’ intention to remain. The better the practice environment was (higher PES‐NWI scores), the more nurses intended to remain in their hospitals, as with previous studies (Gardner, Thomas‐Hawkins, Fogg, & Latham, 2007; Ogata et al., 2011). The relationships seem to reflect that the PES‐NWI was developed based on the characteristics of “magnet hospitals” that draw nurses to them.

### 5.3 Limitations and future research implications

This study has several limitations. First, a self‐report questionnaire cannot distinguish the influence of response bias. Second, the study included only nurses working in the following setting: a non‐university hospital, located in a large city, with more than 50 beds. These participant hospitals may be more motivated to improve the practice environment, because the competition to hire nurses is more acute for city hospitals. Last, the relationships between the nursing practice environment and patient outcomes were not tested, although the environment is thought to substantially affect the quality of care. Future study is needed to consider variations in hospital characteristics, such as urban vs rural settings and to investigate the relationships between the nursing environment and patient outcomes.

## 6 CONCLUSIONS

The Japanese version of the PES‐NWI showed acceptable reliability and validity for hospital nurses in Japan. Studies that involve other nursing populations, such as university hospitals, are needed to confirm these findings. At the same time, many comparative studies using the PES‐NWI internationally as well as domestically are a necessary component in identifying solutions to nurse shortages.

## CONFLICT OF INTEREST

No conflict of interest has been discovered by the authors.

## AUTHOR CONTRIBUTIONS

All authors have agreed on the final version and meet at least one of the following criteria (based on those recommended by the ICMJE):

- substantial contributions to conception and design, acquisition of data, or analysis and interpretation of data
- drafting the article or revising it critically for important intellectual content.

## References

[nop2148-bib-0001] Aiken, L. H. , Clarke, S. P. , Sloane, D. M. , Lake, E. T. , & Cheney, T. (2008). Effects of hospital care environment on patient mortality and nurse outcomes. Journal of Nursing Administration, 38(5), 223 10.1097/01.NNA.0000312773.42352.d7 18469615 PMC2586978

[nop2148-bib-0002] Anzai, E. , Douglas, C. , & Bonner, A. (2014). Nursing practice environment, quality of care and morale of hospital nurses in Japan. Nursing & Health Sciences, 16(2), 171–178. 10.1111/nhs.12081 23855754

[nop2148-bib-0003] Buchan, J. , & Calman, L. (2005). The global shortage of registered nurses: An overview of issues and actions. International Council of Nurses. Retrieved from http://www.icn.ch/images/stories/documents/publications/GNRI/Global_Shortage_of_Registered_Nurses_Executive_summary.pdf.

[nop2148-bib-0004] Chiang, H. Y. , & Lin, S. Y. (2009). Psychometric testing of the Chinese version of nursing practice environment scale. Journal of Clinical Nursing, 18(6), 919–929. 19017371 10.1111/j.1365-2702.2008.02433.x

[nop2148-bib-0005] Cho, E. , Choi, M. , Kim, E. Y. , Yoo, I. Y. , & Lee, N. J. (2011). Construct validity and reliability of the Korean version of the practice environment scale of nursing work index for Korean nurses. Journal of Korean Academy of Nursing, 41(3), 325–332. (in Korean). 10.4040/jkan.2011.41.3.325 21804341

[nop2148-bib-0006] Fuentelsaz‐Gallego, C. , Moreno‐Casbas, M. T. , & Gonzalez‐Maria, E. (2013). Validation of the Spanish version of the questionnaire Practice Environment Scale of the Nursing Work Index. International Journal of Nursing Studies, 50(2), 274–280. 10.1016/j.ijnurstu.2012.08.001 22944284

[nop2148-bib-0007] Gabriel, A. S. , Erickson, R. J. , Moran, C. M. , Diefendorff, J. M. , & Bromley, G. E. (2013). A multilevel analysis of the effects of the Practice Environment Scale of the Nursing Work Index on nurse outcomes. Research in Nursing & Health, 36(6), 567–581. 10.1002/nur.21562 24122833

[nop2148-bib-0008] Gardner, J. K. , Thomas‐Hawkins, C. , Fogg, L. , & Latham, C. E. (2007). The relationships between nurses’ perceptions of the hemodialysis unit work environment and nurse turnover, patient satisfaction and hospitalizations. Nephrology Nursing Journal, 34(3), 271. 17644871

[nop2148-bib-0009] Hanrahan, N. P. , Aiken, L. H. , McClaine, L. , & Hanlon, A. L. (2010). Relationship between psychiatric nurse work environments and nurse burnout in acute care general hospitals. Issues in Mental Health Nursing, 31(3), 198–207. 10.3109/01612840903200068 20144031 PMC2856615

[nop2148-bib-0010] Hooper, D. , Coughlan, J. , & Mullen, M. (2008). Structural equation modelling: Guidelines for determining model fit. Electronic Journal of Business Research Methods, 6(1), 53–60.

[nop2148-bib-0011] Kubo, Y. , Nagamatsu, Y. , Yakeyama, Y. , Anan, A. , Kawamoto, R. , Kanayama, M. , & Murase, C. (2007). An investigation of factors affecting job satisfaction among psychiatric nurse ‐ focussed on stress‐coping and character tendency. Journal of University of Occupational and Environmental Health, 29(2), 169–181. (in Japanese). 10.7888/juoeh.29.169 17582989

[nop2148-bib-0012] Kubo, M. , & Tao, M. (1994). Burnout among nurses: The relationship between stresses and burnout. Japanese Journal of Experimental Social Psychology, 34(1), 33–43. (in Japanese). 10.2130/jjesp.34.33

[nop2148-bib-0013] Lake, E. T. (2002). Development of the practice environment scale of the nursing work index. Research in Nursing & Health, 25(3), 176–188. 10.1002/(ISSN)1098-240X 12015780

[nop2148-bib-0014] Laschinger, H. K. S. , & Leiter, M. P. (2006). The impact of nursing work environments on patient safety outcomes: The mediating role of burnout engagement. Journal of Nursing Administration, 36(5), 259–267. 10.1097/00005110-200605000-00019 16705307

[nop2148-bib-0015] Leiter, M. P. , & Laschinger, H. K. S. (2006). Relationships of work and practice environment to professional burnout: Testing a causal model. Nursing Research, 55(2), 137–146. 10.1097/00006199-200603000-00009 16601626

[nop2148-bib-0016] Liu, K. , You, L. M. , Chen, S. X. , Hao, Y. T. , Zhu, X. W. , Zhang, L. F. , & Aiken, L. H. (2012). The relationship between hospital work environment and nurse outcomes in Guangdong, China: A nurse questionnaire survey. Journal of Clinical Nursing, 21(9–10), 1476–1485. 10.1111/j.1365-2702.2011.03991.x 22380003 PMC3392025

[nop2148-bib-0017] Manojlovich, M. (2005). Linking the practice environment to nurses’ job satisfaction through nurse‐physician communication. Journal of Nursing Scholarship, 37(4), 367–373. 10.1111/j.1547-5069.2005.00063.x 16396411

[nop2148-bib-0018] Manojlovich, M. , & Laschinger, H. (2007). The nursing worklife model: Extending and refining a new theory. Journal of Nursing Management, 15(3), 256–263. 10.1111/j.1365-2834.2007.00670.x 17359425

[nop2148-bib-0019] Maslach, C. , & Jackson, S. E. (1981). The measurement of experienced burnout. Journal of Organizational Behavior, 2(2), 99–113. 10.1002/(ISSN)1099-1379

[nop2148-bib-0020] McClure, M. L. , Poulin, M. A. , Sovie, M. D. , & Wandelt, M. A. (1983). Magnet hospitals. Attraction and retention of professional nurses. American academy of nursing. Task force on nursing practice in hospitals. Kansas City, MI: American Nurses’ Association. 6551146

[nop2148-bib-0022] Motomura, Y. , & Yatsushiro, R. (2010). Factors relating to nurse burnout. Japanese Journal of Occupational Medicine and Traumatology, 58(3), 120–127. (in Japanese).

[nop2148-bib-0023] Nakagaki, A. (2011). Midwives’ time perspective and its relationship to job satisfaction examined from the viewpoint of life span development. Japanese Journal of Maternal Health, 52(2), 294–302. (in Japanese).

[nop2148-bib-0024] Narita, M. , & Ishii, N. (2015). The relationship between a nurse's nursing practice environment and job satisfaction: Exploring the experience of nurses 2 to 3rd after graduation. Health Sciences Bulletin Akita University, 23(2), 35–47. (in Japanese).

[nop2148-bib-0025] Ogata, Y. , Nagano, M. , & Akanuma, T. (2008). Translating “The Practice Environment Scale of the Nursing Work Index (PES‐NWI)” into Japanese. Journal of School of Nursing, Chiba University, 30, 19–24. (in Japanese).

[nop2148-bib-0026] Ogata, Y. , Nagano, M. , Fukuda, T. , & Hashimoto, M. (2011). Job retention and nursing practice environment of hospital nurses in Japan applying the Japanese version of the Practice Environment Scale of the Nursing Work Index (PES‐NWI). Japanese Journal of Public Health, 58(6), 409–419. (in Japanese). 21970075

[nop2148-bib-0027] Ogata, Y. , Nagano, M. , Nishioka, M. , Akanuma, T. , Uchida, A. , Yamana, T. , & Hashimoto, M. (2010). Preliminary study of the reliability and validity on the Practice Environment Scale of the Nursing Work Index, PES‐NWI (Japanese version). Journal of the Japan Society for Healthcare Administration, 47(2), 69–80. (in Japanese).

[nop2148-bib-0028] Ozaki, F. , & Tadamasa, T. (1988). A study on the measurement of nurses’ job satisfaction with their work situation in Japan : Trying to apply a questionnaire developed by Stamps and others. Bulletin of Osaka Prefectural College of Nursing, 10(1), 17–24. (in Japanese).

[nop2148-bib-0029] Parker, D. , Tuckett, A. , Eley, R. , & Hegney, D. (2010). Construct validity and reliability of the Practice Environment Scale of the Nursing Work Index for Queensland nurses. International Journal of Nursing Practice, 16(4), 352–358. 10.1111/j.1440-172X.2010.01851.x 20649666

[nop2148-bib-0030] Patrician, P. A. , Shang, J. , & Lake, E. T. (2010). Organizational determinants of work outcomes and quality care ratings among Army Medical Department registered nurses. Research in Nursing & Health, 33(2), 99–110. 10.1002/nur.20370 20151409 PMC2969846

[nop2148-bib-0031] Pedro‐Gómez, D. , Morales‐Asencio, J. M. , Sesé‐Abad, A. , Bennasar‐Veny, M. , Pericas‐Beltran, J. , & Miguélez‐Chamorro, A. (2012). Psychometric testing of the Spanish version of the practice environment scale of the nursing work index in a primary healthcare context. Journal of Advanced Nursing, 68(1), 212–221. 10.1111/j.1365-2648.2011.05730.x 21711384

[nop2148-bib-0032] Stamps, P. L. , Piedmont, E. B. , Slavitt, D. B. , & Haase, A. M. (1978). Measurement of work satisfaction among health professionals. Medical Care, 16(4), 337–352. 10.1097/00005650-197804000-00006 651399

[nop2148-bib-0033] Steiger, J. H. (2007). Understanding the limitations of global fit assessment in structural equation modeling. Personality and Individual Differences, 42(5), 893–898. 10.1016/j.paid.2006.09.017

[nop2148-bib-0034] Tao, M. , & Kubo, M. (1996). Burnout: Theory and practice. Tokyo: Seishinshobo Press. (in Japanese).

[nop2148-bib-0035] Tei‐Tominaga, M. , Tsuchiya, M. , & Sato, F. (2012). Characteristics of the work environment of magnet hospitals and job satisfaction among nurses in Japan: A cross‐sectional study using multi‐level analysis. Journal of Nursing & Care, S5, 003.

[nop2148-bib-0036] Toyoda, H. (2011). Covariance structure analysis with amos – Structural equation modeling. Tokyo: Tokyo Tosho. (in Japanese).

[nop2148-bib-0037] Ueno, E. , Syoji, Y. , Setsuko, T. , Takashima, S. , & Takama, S. (1999). The influence of nurses’ opner and lonliness on their job satisfaction. Journal of the Nursing Society of the Toyama Medical and Pharmaceutical University, 2, 117–126. (in Japanese).

[nop2148-bib-0038] Warshawsky, N. E. , & Havens, D. S. (2011). Global use of the practice environment scale of the nursing work index. Nursing Research, 60(1), 17 10.1097/NNR.0b013e3181ffa79c 21127450 PMC3021172

